# Evolution of Microbial Quorum Sensing to Human Global Quorum Sensing: An Insight into How Gap Junctional Intercellular Communication Might Be Linked to the Global Metabolic Disease Crisis

**DOI:** 10.3390/biology5020029

**Published:** 2016-06-15

**Authors:** James E. Trosko

**Affiliations:** Department of Pediatrics/Human Development, College of Human Medicine, Michigan State University, East Lansing, MI 48824, USA; james.trosko@hc.msu.edu; Tel.: +1-517-884-2053

**Keywords:** quorum sensing, gap junctions, stem cells, epigenetic mechanisms, biological and cultural evolution, symmetrical cell division, Warburg hypothesis, Barker Hypothesis

## Abstract

The first anaerobic organism extracted energy for survival and reproduction from its source of nutrients, with the genetic means to ensure protection of its individual genome but also its species survival. While it had a means to communicate with its community via simple secreted molecules (“quorum sensing”), the eventual shift to an aerobic environment led to multi-cellular metazoan organisms, with evolutionary-selected genes to form extracellular matrices, stem cells, stem cell niches, and a family of gap junction or “connexin” genes. These germinal and somatic stem cells responded to extracellular signals that triggered intra-cellular signaling to regulate specific genes out of the total genome. These extra-cellular induced intra-cellular signals also modulated gap junctional intercellular communication (GJIC) in order to regulate the new cellular functions of symmetrical and asymmetrical cell division, cell differentiation, modes of cell death, and senescence. Within the hierarchical and cybernetic concepts, differentiated by neurons organized in the brain of the *Homo sapiens*, the conscious mind led to language, abstract ideas, technology, myth-making, scientific reasoning, and moral decision–making, *i.e.*, the creation of culture. Over thousands of years, this has created the current collision between biological and cultural evolution, leading to the global “metabolic disease” crisis.

“ …, since the mechanism of cell communication itself is universal in biology, in keeping with a Kuhnian paradigm shift. This approach may even elucidate the nature and evolution of consciousness as a manifestation of the cellular continuum from unicellular to multicellular life. We need such a functional genomic mechanism for the process of evolution if we are to make progress in biology and medicine.” John S. Torday, Prospect Biol. Med. 56: 455, 2014.

## 1. Introduction: A Biological Rosetta Stone: Understanding the Roles of Cell-Cell Communication during the Evolution of All Living Organisms in Their Struggles for Life and Reproduction in an Ever-Changing Physical, Chemical, Social, and Cultural World

Most of us as young, inexperienced scientists were told that we must “Tell them what you are going to tell them; tell them; and in the end, tell them what you told them!” Therefore, the current objective is to try to link the biological evolution of the first primitive single-celled organism’s ability to communicate, namely via “quorum sensing,” to a more complex set of genes, structures, and processes in non-human multi-cellular organisms—ultimately to a very unique family of genes/structures and functions, namely the connexin genes and gap junctional intercellular communication (GJIC). These genes have made possible human consciousness, abstract thinking, creation of symbols, language, technologies, and the ability to value because they afforded a society of adherent cells that communicate with each other to coordinate and regulate electronic and metabolic synchronization, homeostatically. This current evolution of the metazoan’s ability to communicate for the purpose of survival and reproduction confronted a historically unique health/disease problem. Millions of years of slow biological evolution to select genes needed to obtain energy for life and reproduction in a rather slowly changing physical environment, gave way to rapidly changing “cultural evolution,” which involves all aspects of the elements affecting food sources [1,2].

In trying to explain a complex potential hypothesis for the evolution of life, without the need for divine intervention or “vital forces,” using only scientific knowledge—which, at worst, could be dead wrong, and, at best, is always incomplete but self-correcting—the shorthand message is: the sequestration of the first living microorganism from the primordial “soup” of nutrients, which provides energy for life and reproduction, was the formation of a semi-permeable membrane to provide a sense of “self” and the means to communicate with its progeny. While the start of this analysis must begin with very few facts about the first set of molecular events to create the first living, self-replicating cell, it is clear that many independent events must have occurred to create a small space in that vast primordial sea, in which the “inside” was separated from the “outside.” This implies the formation of a semi-permeable membrane. In addition, that “inside space” had to facilitate the movement of ions/small molecules, in and out of that space, while at the same time allowing for the creation of self-replicating molecules that code for all the components to allow this unique separated space to maintain “homeostatic control of all the necessary biochemical functions” to allow this cell to generate energy for life and reproduce its survival machinery in an ever-changing world.

It is not to be underestimated that our understanding of those early events is, today, still unknown. However, it must have happened because before that “moment” in history, there was no living organism. In addition, in keeping with this analysis, it occurred within natural physical and chemical scientific processes, not by “divine intervention” or by “vital forces.”

From the beginning of that moment to this day, when there are seven billion human beings on earth surrounded by untold numbers of other micro- and macro-species, an environment is being depleted of vital factors needed for basic survival, as well as polluted with the degradable and undegradable detritus of the unconscious and conscious, culture-building *Homo sapiens*. It is necessary to keep in mind the continuous and intimate interconnectedness of life to ponder how the current situation on Earth, including the continuous loss of species and their state of health, might be associated with the evolution of communication: first “quorum-sensing” [3] between micro-organisms, then chemical communication between unconscious plants, and on to various forms of primitive “languages” of multi-cellular metazoans (visual-based body language and body phenotypes; tactile, sound or voice communication, *etc.*) and consciousness of one’s individual consciousness via gap junctional intercellular communication (GJIC). While it might seem outrageous to single out the family of connexin or gap junction genes from the approximately 25 thousand genes in the human genome that led to (a) symbols of conscious objects; (b) translation of those abstractions so a spoken language could be communicated; (c) the transformation of those abstract symbols or ideas into technologies; and (d) finally, the ability to decide or “value” whether to apply that technology for life and reproduction [4], the premise is based on the fact that, without those early acquired genes, none of the differentiated cells, such as neurons and the functional brain, would exist. This concept of *Homo sapiens’* state of evolution lets us see that “culture” has emerged as an evolutionary process via cybernetic and hierarchical principles [5,6].

The thesis of this complex view of evolution, then, tries to link “quorum sensing” to primitive chemical and non-language-based communication to conscious, culturally-dependent forms of language-based communication to a current form of social networking via high-tech communication (Twitter, Facebook, Instagram, e-mail, *etc.*). This could lead to a selective “global consciousness.” Some might argue that this attempt to link, via evolution, “microbial quorum sensing” to *Homo sapiens’* ability to use a culturally-dependent formation of scientific and technological knowledge to shape a new form of “global consciousness” is but a delusional, misuse of human consciousness. However, this is the thesis that will be used in order to examine one major crisis that any organism must face in an ever-changing physical, chemical, social, and cultural environment. In the case of human beings’ struggle for life and reproduction for the individual and species survival, there is an impeding collision of the glacially slow pace of biological evolution of genes needed for survival in the current, inevitably changing environment with the laser-speed cultural evolutionary impact on the physical, chemical, and psycho-social environments that impact on those genes inherited over millions of years [1,2]. Human beings no longer live in a “jungle” environment, but a “concrete” environment.

In brief, as a part of “telling what I’m about to tell you,” I am going to hypothesize that the biological evolution of extracellular matrix molecules, stem cells, a stem cell-low oxygen-niche, and a family of highly evolutionarily-conserved genes (*i.e.*, the connexin genes) allowed for the formation of both consciousness and the “consciousness of one’s consciousness” (“*As a result of a thousand million years of evolution, the universe is becoming conscious of itself, able to understand something of its past history and its possible future.”—Julian Huxley*). This led to the creation of cultural evolution, which is on a dangerous collision course with the biological evolution of those genes that led to the widespread human ethnic groups to survive on the foods of their original existence. Because of the current diaspora of these ethnic groups and their genes and foods, as well as pluralistic values due to our cultural evolution, the use and misuse of scientific, technological, and non-scientific views of human nature [7] are affecting the very physical environment on which our genes depend on for a normal, healthy, and high-quality life. From “quorum sensing” of single-celled organisms to global “consciousness,” via culturally created Internet communications technology, we now have the potential for “*human quorum-sensing.*”

## 2. The Transition from an Anaerobic to an Aerobic Environment Forced the Selection from Quorum Sensing of Single-Celled Organisms and to Gap Junctional Intercellular Communication Needed for Metazoans

It has to be stated upfront that this subtitle does not imply a direct, linear selection of the family of gap junction genes that led to the metazoans and *Homo sapiens*, but that, during evolution, a number of unknown (to date) gene acquisitions, on which the connexin genes and the function of gap junctions depended, occurred. In other words, the connexin genes and the functional gap junction did not just appear out of nowhere and, all of a sudden, multicellular organisms with stem cells, stem cell niches, and differentiation resulted. There are many gaps of knowledge, during the evolutionary selection of this fundamental biological process of gap junctional intercellular communication, of all the other genes crucial to the function and regulation of these connexin genes and the proteins of the gap junctions.

After thriving in an oxygen-poor environment, anaerobic single cell organisms found themselves in a toxic oxygen-rich atmospheric and aqueous environment, created by the emergence of cyanobacteria, which were able to convert energy from sunlight to convert CO_2_ and H_2_O into sugars and other carbohydrates by photosynthesis and generating oxygen as a byproduct [8,9]. While this new oxygenated environment was toxic to these anaerobic single-celled organisms, it provided stress for the selection of new life forms that could cope with this oxygenated environment. Obviously, the paleochemistry of the earth’s early environment would not have allowed our current life forms to have existed. The elevated temperatures, available chemicals (as potential nutrients), radiation levels, gravity, and atmospheric gases set the limits to which evolutionary forces selected modern forms of life to exist. New genes and cellular options needed for existence started to emerge [10]. A new means for generating energy from nutrients came about during a relatively stable short range of extremes in temperature, specific ratios of ambient gases, the change in seasons, and diurnal cycles of light. This helped to select new genes (mutational mechanisms) and methods to regulate these genes (epigenetic mechanisms or the molecular means to regulate sets of genes at the transcriptional, translational, and posttranslational levels).

Since anaerobic single-celled organisms metabolized sugars via glycolysis to produce ATP for living organisms’ source of life and reproduction (fermentation), the very inefficient process limited the cells’ adaptive options. Although scientific information as to the origin of the evolutionary appearance of the fusion of the mitochondrion and early pre-metazoan cell is limited, this symbiotic fusion of the mitochondrion with a uni-cellular organism led to dramatic new adaptive options for a cell to survive in this oxygenated environment [11].

First and foremost was this new type of cell’s ability to be extremely efficient in the production of ATP (oxidative phosphorylation) [9]. This afforded the new cell options for adaptive behavior. To put this in perspective, single-celled organisms existed as a population of “free-floating” individuals. Even biofilm-single-celled organisms [12], while existing in a two-dimensional coexisting population, could not “communicate” with each other as would the new metazoan as a three-dimensional social collection of mitochondria-containing cells. Not only would these new cell types be able to generate ATP more efficiently to enable the cell to adapt to changing environments, but also, because of one of the “byproducts” of oxygen metabolism, these cells produced a molecule that could only be produced with oxygen, a sticky cellular excreted collagen-type molecule [13,14]. This allowed these mitochondrial-energy-producing cells to stick together via these extracellular molecules. However, that alone was not sufficient for what was to follow, namely a means for the differential collection of socially communicating cells, utilizing the primitive forms of secreting small extra-cellular signaling molecules (hormones, cytokines, growth factors) that triggered intra-cellular signaling to (a) selectively regulate the expression of selective sets of genes and (b) modulate (increase or decrease) a new form of direct transfer of ions and small molecular weight regulatory molecules via a membrane-associated protein channel and intercellular communication via the gap junction.

Although more information is needed to study the evolutionary history of the origin of the highly evolutionarily conserved family of genes [15,16], it appears to be early in the evolution of multi-cellularity [17,18] and the 3D nature of the metazoan [19]. The transition of the single-celled organism to the first multi-celled organism had to be assisted by a number of newly selected adaptive phenotypes coded by new genes and epigenetic mechanisms. Obviously, the first new phenotype had to be “growth control.” While the metazoan cell had to regulate growth as does the single-celled organism, when the appropriate nutrients, temperatures, atmospheric gases, and radiation levels limited cell proliferation, the social organization of cells required a new means of control in this multicellular organism because uncontrolled growth would end up as a tumor. Harry Eagle termed this type of metazoan growth control “contact inhibition” [20], which occurs when two cells in direct physical contact with each other to send signals to control their ability to proliferate. Cell adhesion, extracellular matrix molecules, and gap junctional intercellular communication would be shown to be required for *progenitor* cells (more on the growth control of *stem* cells later).

The second new metazoan phenotype had to be a new means of regulating the selective sets of the total genomic information found in all cell types of the metazoan (“*epigenesis*”). This required new genes to regulate gene expression or the genes to encode the epigenetic biochemical mechanisms to regulate gene expression at the *transcriptional* level (methylation/ethylation of DNA & histone molecules); the *translational* levels (*i.e.*, splicing mechanisms); and *posttranslational* modification of coded proteins (e.g., phosphorylation of proteins; micro-RNAs). This allowed the metazoan cell, by the expression of certain sets of genes found in all cells, to be phenotypically unique, namely they can be “differentiated” into differentially functioning cells.

The third new phenotype is that of unique, gene-regulated means of cell death, such as “apoptosis.” During the development of a metazoan, cells selectively remove damaged or non-adaptive differentiated cells during specific periods of development. The fourth new phenotype that was selected was the induction of “senescence.”

**Figure 1:**
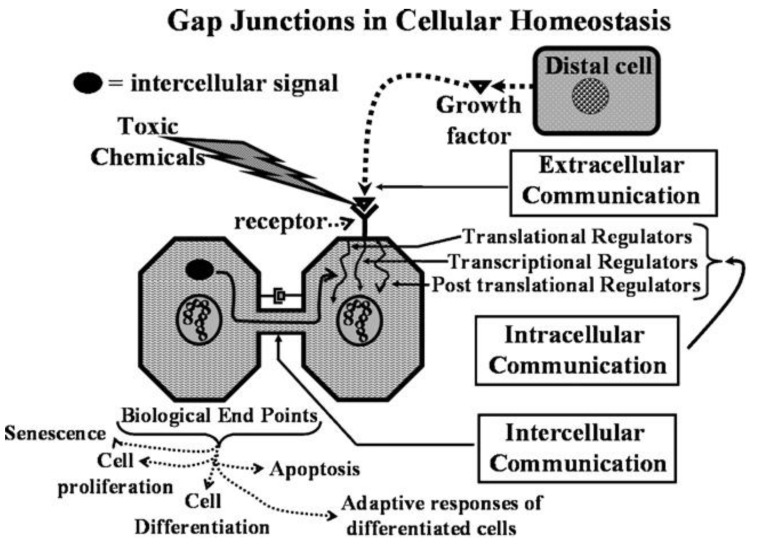
Gap junctions in cellular homeostasis. Extracellular signals, such as growth factors, cytokines, hormones, toxicants, extra-cellular matrices, and cell adhesion molecules, that vary for each cell type (adult stem cell, progenitor, and terminally differentiated), interact with receptor-dependent or receptor-independent targets, which then activate intracellular signal transduction pathways that induce the transcription of genes through activated transcription factors. These specific intracellular pathways operate under cascading systems that cross-communicate with each other in controlling the expression of genes that direct the proliferation, differentiation, and apoptosis of cells within a tissue. These multiple intracellular signaling check points are further modulated by intercellular signals traversing gap junctions, thereby maintaining the homeostatic state of a tissue. Abnormal interruption of these integrated signaling pathways by food-related and environmental toxins/toxicants will disrupt the normal homeostatic control of cell behavior (Permission granted from *Toxicology* 2010, 270, 18–34 [22]).

This new evolutionary phenomenon integrated extracellular, intracellular, and gap junctional intercellular communication in a tightly orchestrated “system” or cybernetically regulated whole [5,6,21] (Figure 1).

## 3. Biological Evolution of the Gap Junctional Intercellular Communication with the Appearance of Stem Cells and Cell Differentiation

Another major gap in our scientific knowledge is represented by the question “*Which came first, the connexin genes and gap junctional intercellular communication or the stem cells?*” This classic “chicken or egg” problem will go without comment, except it seems these two biological features are inexorably linked in the formation of the metazoan and the process of differentiation.

Together with the four new phenotypes that previously appeared with the appearance of the metazoan, the most important new phenotype to appear in the metazoan was the formation of germ and somatic stem cells [23]. These new cell types share one form of cell division with its single-celled evolutionary precursor, namely the ability to divide, *symmetrically*, to form two cells with the ability to divide indefinitely. However, the metazoan stem cells also have the ability, under specific external conditions, to divide *asymmetrically.* While it is assumed that under these conditions, one daughter cell is destined to terminally differentiate, the other “daughter” maintains “stemness” and the ability to maintain an infinite ability to this stemness state. While this concept is being challenged—namely, stem cells are “immortal” and do not “age” [24]—for practical purposes, in the absence of a universally accepted interpretation of the “stemness” state, it will be assumed that those cells, having been characterized as having the ability to divide both symmetrically or asymmetrically, do have *extended* life spans compared to their progenitor offspring. More will be discussed on this matter, on the understanding that both *germinal and somatic stem cells* could accrue mutations via “errors in replication” if stimulated to divide too frequently. This is one reason why most stem cells seem to divide infrequently [25]: to minimize the formation, but not the total elimination, of mutations in both types of stem cells.

**Figure 2:**
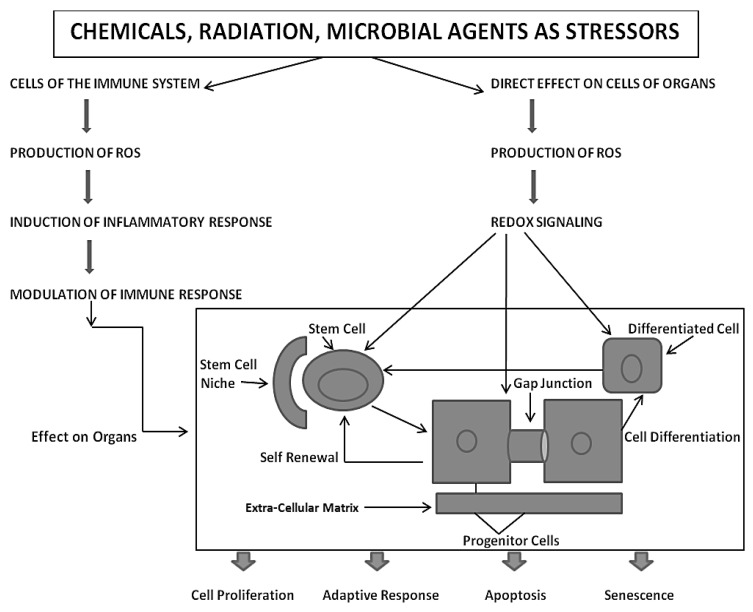
This diagram depicts the homeostatic interaction of extra- and intercellular signaling between the three types of cells within a tissue, triggered by endogenous and/or exogenous factors, which can alter, differentially, intracellular signaling and gene expression in the stem, progenitor, and terminally differentiated cells. In the context of the whole organism, an external stressor will affect cells of multiple organs, including the immune cells, which, in turn, respond by producing various inflammatory soluble molecules that can differentially affect the various cell types of the directly exposed stressor.

All of these new phenotypes associated with the appearance of the metazoan cell, which socially and functionally organize into 3D structures, can form differentiated tissues [18,26,27] and have new cellular functions, such as senescence and apoptosis, and can help to maintain the genetic integrity of its genome for the species and its individual owner. Therefore, the integration of these various forms of cell communication is carefully orchestrated in the developing, adolescent, mature, and geriatric individual, as shown in Figure 2.

From this integrated concept of how these three forms of cell-cell communication help to maintain homeostatic control of the main functions of a cell during the development, adolescence, maturation, and geriatric states of life of the metazoan, including the human being, one can see how either programmed or untimely disruption of this highly orchestrated communication network can lead to adaptive or non-adaptive physiological/toxicological responses, respectively. It cannot be over-emphasized that, since most of the cells in the metazoan are communicating with each other by extracellular or gap junctional intercellular communication mechanisms, the intracellular signaling pathways, affected by either extra- or gap junctional mechanisms, will determine the affected cells’ cellular responses. These signals are either produced by normal endogenous chemicals, induced by development stages, diurnal rhythms, growth, wound healing and tissue repair/removal, or by the inevitable interactions with exogenous signals from foods, drugs, pollutants, and stress [23]. It is this dynamic complexity of interaction of different mixtures, concentrations, timings, individual genetics, genders, and developmental stages that can modify the net result of that interaction.

**Figure 3:**
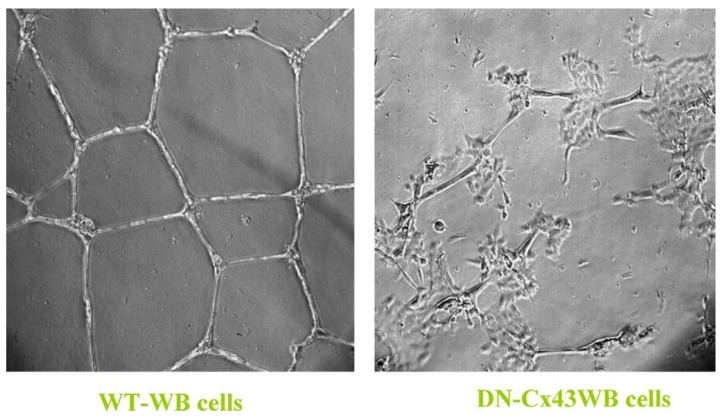
In these two panels, when the wild-type (WT) rat liver oval cells are placed on Matrigel, within 24 h the cells organize into a well-structured network. On the other hand, when a dominant negative (DN) Cx43 is transected into the wild-type rat liver WB cells and then placed on Matrigel, the cells lose their ability to form these organized networks because their gap junctions become dysfunctional. It is clear that some “organizing” signal is necessary to cause the individual cells to form these networks. Permission granted from De Feijter, *et al*., [29].

Finally, the role of gap junctional communication is ultimately the critical evolutionary contribution to all the attributes of being human, including those brain/mind functions that seem unique to being a conscious, creative, and decision-making creature [28]. At this point, a simple observation to make is that the normal function of gap junctional intercellular communication is fundamental to the development of becoming a 3D metazoan. This is illustrated in Figure 3.

When a normal rat liver oval cell, which expresses the normal, functioning connexin43 gap junction protein, is randomly placed on a Matrigel substrate *in vitro*, it will form, within 24 h, a beautiful, regular network structure (Panel A). In addition, growing stem cells in three-dimensional cultures can generate organoids, such as mini-breast tissue [26] mini-guts, optic-cups, and mini-brains [27].

However, if these cells are genetically engineered to express a dominant negative connexin43 gene, the gap junctions are no longer functional. When these DN-Cx43 cells are placed on Matrigel in an identical fashion to their normal counterparts, these beautiful network structures are not formed. This implies that signals that normally pass through these gap junctions are responsible for allowing the expression of genes in these cells to help start the normal development process of making the liver. Noting that all phenotypes are the result of gene and environmental interactions, not only can one disrupt gap junctional communication genetically (as is this case with a DN-Cx43 genetically-engineered procedure or hereditary human syndromes with inherited connexin genes [30]), but also by disruption of GJIC by endogenous (growth factors, cytokines, hormones) or exogenous factors (drugs, pesticides, pollutants) [31].

## 4. The Normal or Dysfunctional Consequences of Cell-Cell Communication in Various Cell Types

Based on the assumption that the evolutionary transition from the single-celled organism, living in an anaerobic environment, to the metazoan, having acquired (a) all the genetic blueprints to form germinal and somatic stem cells and (b) an integrated cell-cell communication system to homeostatically control cell proliferation, cell differentiation, apoptosis, and senescence under both anaerobic and aerobic conditions, one must put the mechanisms of mutagenesis and epigenesis in perspective by considering the ability of both the individual metazoan organism and its species to survive in an ever-changing environment. With the acquisition of genes to perform oxidative phosphorylation for the maximum production of ATP for energy, the need was to provide a reduced oxygen microenvironment for the stem cell, which exists near the high oxygen microenvironment of its differentiated daughters [32,33,34].

The extremely complex co-evolution of genes was needed to cope with the negative side-effects of oxidative phosphorylation, namely the generation of many reactive oxygen species (ROSs)/reactive nitrogen species (RNSs) [33], as well as to utilize them as adaptive signal transducers of gene regulators [35,36]. Since a balance of mutations and epigenetic alterations depends on the integrity of genomic and mitochondrial DNA, genetic mechanisms had to be selected that protected DNA from these highly reactive ROSs. This had to include a number of genes that protected genomic and mitochondrial DNA from ROS-induced macromolecular damage (endogenous antioxidants) and repaired the inevitable damage that might occur during “*errors of DNA repair*,” such as in the skin-cancer-prone “xeroderma pigmentosum” genetic syndrome [37,38,39]. It is here that that one must recognize that mutations can occur without any DNA damage via “*errors in DNA replication*,” such as in the cancer-prone Blooms genetic syndrome [40].

Also, with the evolutionary selection of extracellular matrix molecules to help create a niche or low-oxygen microenvironment for stem cells, which would minimize germ and somatic stem cell genomic DNA damage from ROSs, this helped to create a stem cell physiology similar to the single cell’s physiology in an anaerobic environment, in order to maintain its undifferentiated or “stemness” state [8,10,34,41]. Protecting and repairing genomic and mitochondrial DNA is a critical function for the survival and health of the individual organism and for sustaining the species. A “healthy balance” between a perfect protection of the genomic integrity and a very sloppy protective and repair system is needed to be adaptive in an inevitable changing physical environment. Too many genomic/epigenetic changes, as well as too few, would be non-adaptive in a changing environment.

Therefore, the selection for these balanced protective and repair systems allowed for the frequency of somatic and germinal mutations to be sufficient for the individual to survive long enough to reproduce and allow for the reproductive survival of their offspring. If the mutation frequencies were too high in either the germinal or somatic stem cells, the individual would suffer mutation-related diseases, which could risk their ability to reproduce and sustain the survival of its species.

This brings up the notion, from an evolutionary perspective, that the stem cells of an individual had to develop means to be more resistant to toxic environmental factors than its progenitors and differentiated daughters. If, when a metazoan is exposed to a toxic agent, the stem cells were of equal sensitivity to these agents as their progenitor or differentiated daughters, then the chances of recovery and survival of individual would be jeopardized. Observations to date seem, in general, to support the idea that some somatic organ-specific stem cells appear to be more resistant to physical and chemical toxicants [42]. One of the gene sets responsible for this resistance to toxic chemicals appears to be the expression of the drug transporter genes in stem cells, which is absent in the progenitor or differentiated cells [43].

Another factor in the differential reaction of stem cells, compared to their progenitor and differentiated daughters, is the number of mitochondria in the stem cells, where most of the ROSs are generated [44,45,46,47,48]. This would suggest that both the potential genotoxic and epigenetic effects of the ROSs would be much more limited than in the differentiated daughters. Another way of understanding the differential sensitivity of stem cells, progenitors, and differentiated cells is to keep in mind the fact they all express different genes and have different internal physiology and reactions to exposure to exogenous chemicals/physical agents. One example would be the hepatocyte cells of the mature liver. These cells perform the function of the liver as the major toxic filter organ. There were several evolutionary strategies to make these cells more efficient as detoxifiers. One way was to mutate the detoxifying-related genes to be hyperactive. Another strategy was to amplify the number of detoxification genes. Note that in the mature liver the hepatocyte population includes diploid, tetraploid, and octaploid hepatocytes. In other words, once the diploid hepatocyte cell is derived from its liver stem cell, it can be stimulated to replicate its DNA, but it fails to go through cytokinesis, producing a tetraploid cell. This cell can also be stimulated to replicate its DNA, resulting in a cell that cannot go through cytokinesis. Therefore, one ends up with an octaploid cell with eight copies of the genes responsible for detoxification.

The understanding of the evolution of stem cells—which metabolize glucose via glycolysis or fermentation, have few mitochondria, and can be induced to differentiate, generate many mitochondria, and metabolize via oxidative phosphorylation—has practical implications for our current understanding of how epigenetic mechanisms play a major role in the toxic effects after exposure to physical and chemical agents [49]. Understandably, the role that mutagenic agents play in heredity and somatic diseases is real and important. However, because of the misinterpretation of *in vitro* and *in vivo* assays to measure mutations, especially after chemical exposures that gave rise to birth defects, cancer, immune responses, and reproductive and neurological pathologies, it has been assumed that these agents caused the mutations found in the diseased tissues [50,51].

When it was shown that many chemicals in organs of animals could be metabolized to form highly reactive electrophiles [52], and that they were formed in differentiated cells such as hepatocytes, it was generally assumed that the chemical to which an animal or human being was exposed caused the mutation found in an oncogene of a cancer (and some other disease states). However, when the attention of the public was shifted by Rachel Carson’s book *Silent Spring* [53], chemicals, rather than radiation, started to be tested for their potential toxic effects on humans. When the molecules DDT, polybrominated biphenols, TCDDs, phthalates, endocrine disruptors, *etc.*, were tested for “mutagenicity,” using various *in vitro* assays, they were found wanting. On the other hand, using various assays to measure how non-mutagenic, non-cytotoxic chemicals could affect cell-cell communication [31], all of these chemicals, which could lead to birth defects, tumor promotion, immune modulation, and reproductive and neurological dysfunctions, were shown to be “epigenetic toxicants” [54,55,56].

**Figure 4:**
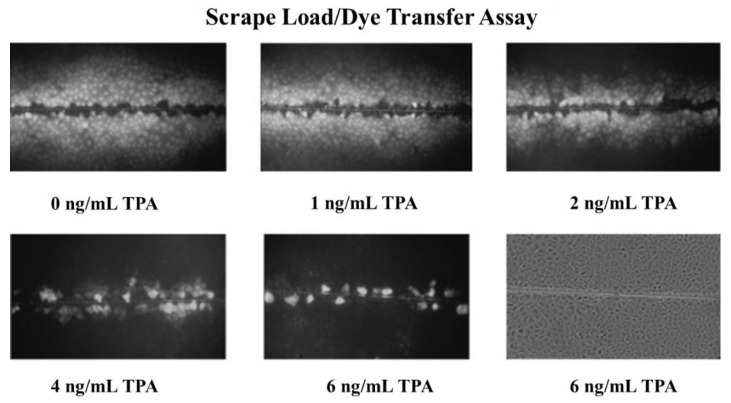
This is a series of *in vitro* measurements of GJIC, using the scrape loading/dye transfer technique. Rat liver WB F344 cells were treated with various concentrations of phorbol ester, TPA, for 15 min; cells were exposed to Lucifer yellow dye, and then scraped with a scalpel. After 3 min, the dye was removed; cells were fixed and subsequently examined under an epifluorescent microscope. As can be seen, there is a clear dose-responsive inhibition of GJIC, as measured by the diffusion of dye from the scrape line. This phenomenon occurs at non-cytotoxic concentrations and it is reversible. (Permission granted from Trosko and Chang [22]).

An example of how a classic non-mutagenic chemical, at non-cytotoxic concentrations, could inhibit gap junctional intercellular communication is shown in Figure 4.

Some general characteristics can be drawn from studies of the epigenetic-acting chemicals, namely they can (a) be species specific, (b) gender specific, (c) developmental stage specific, and (d) threshold dependent; (e) they can act additively, antagonistically, or synergistically with other chemicals; and (f) they are dependent on time of exposure. One should also note that, in the metazoan, since organ-specific stem cells reside with their progenitor and differentiated daughters, these chemicals could modulate non-gap junctional communication (*i.e.*, extracellular) communication between stem cells and their differentiated daughters or the extracellular matrix, thereby causing either disruption of cell proliferation or differentiation homoeostasis. This might be the mechanistic basis for the Barker hypothesis [1,49,57].

In other words, during development of the embryo and fetus, gap junctions were needed for specific tissue-type differentiation. Embryo lethality or fetal development could be disrupted on an organ-specific stage or site. A great example of such a chemical is thalidomide. This chemical was developed because it acted, pharmacologically, as a great sedative [58]. Later, it was shown to be a tragic human teratogen when given to pregnant women during a critical “window” of fetal development. It was also used for treatment of leprosy [59]. Even later, it has been used as an anti-cancer agent because of its anti-angiogenesis properties [60]. Today it is known to modulate gap junctional intercellular communication [61].

To put this phenomenon in perspective, since gap junctional intercellular communication is critical in the regulation of progenitor cells, which express connexin genes and have functional gap junctions, inhibition of gap junction function at any level could cause disruption of cell replication, differentiation, or apoptosis. In the case of stem cells, which do not seem to express the connexin genes or have functional gap junctions, exposure to epigenetic agents that could interfere with the secreted negative growth regulators from their differentiated daughters [62,63,64,65,66] could cause these stem cells to proliferate. On the other hand, exposure to these epigenetic chemicals (either endogenous or exogenous) could cause them to differentiate. Again, it has to be clearly stated that acute modulation of GJIC is an adaptive function. However, inappropriate acute disruption of GJIC function (increased or decreased) at a critical time during development could lead to a pathological consequence (teratogenesis), whereas chronic disruption in the adult could have different types of pathologies, such as tumor promotion [56].

## 5. Role of Adult Stem Cells and Modulated Cell-Cell Communication in the Multi-Stage, Multi-Mechanism Process of Carcinogenesis

To link stem cells and cell-cell communication with the mechanisms of mutagenesis and epigenetic alteration of gene expression, cancer can be taken as a case study. In brief, it will be hypothesized that cancer is the inevitable consequence of mutations and epigenetic mechanisms serving their vital functions in the evolution of the metazoan. Mutations occurring in the germ line that could serve to provide adaptive features for the survival of the species could provide the grist for the start of the multi-state, multi-mechanism process (the “initiation”/“promotion”/“progression” concept) of carcinogenesis [67,68,69]).

While the two extreme hypotheses for the genesis of this carcinogenic process have not yet been universally accepted, namely the *stem cell hypothesis* [70,71,72,73,74] and the “*de-differentiation*” or “*re-programmed*” hypothesis [75], it will be argued that there seems to be some strong evidence that the organ-specific adult stem cell is the origin of the carcinogenic process [76].

**Figure 5:**
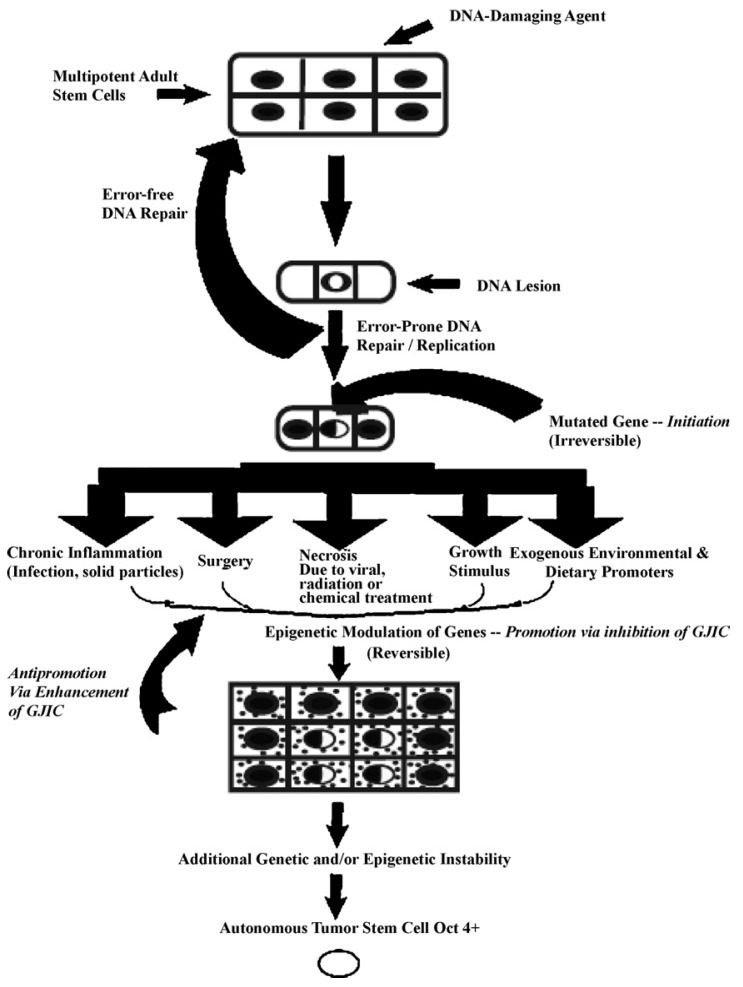
A diagrammatic heuristic scheme to depict the postulated mechanisms of the initiation and promotion phase of carcinogenesis. DNA lesions, induced by physical mutagens or by errors in DNA replication, are substrates in adult stem cells (Oct-4+) that can be fixed if they are not removed in an error-free manner prior to DNA replication. Promotion includes those conditions (*i.e.*, chronic inflammation induced by infectious agents, solid particles; surgery or wounding; necrotic cell death; normal growth stimuli caused by growth factors, hormones; and exogenous epigenetic natural and synthetic molecules), in which a pluripotent, but surviving, initiated adult stem cell (Oct-4+), can escape the non-proliferative state. The build-up of initiated cells allows them to “resist” the anti-mitotic influence of neighboring non-initiated cells. In addition, the changing micro-environment within the growing benign tumor will cause some of the initiated adult stem cells to partially differentiate into cancer non-stem cells. This, together with either addition mutations or stable epigenetic changes, might allow a given initiated adult stem cell to have the autonomous, invasive properties of a malignant cell. Permission granted from Trosko, J.E. and Tai, M.H. [69].

In Figure 5, one proposed hypothesis is presented. In this hypothesis, it is assumed that there exist, in all organs, organ-specific adult stem cells that are needed for growth, tissue replacement, or removal of dead cells. In the case of an inherited germ line mutation, such as in xeroderma pigmentosum and Blooms syndrome (among many other cancers [39,40]), there would be a high risk of “initiating” a single stem cell by affecting a gene or genes that might regulate whether that stem cell can divide symmetrically or asymmetrically. In addition, in an individual conceived with no inherited germ line mutation that would affect the frequency of mutation production, there could be a mutated germline mutation that might affect a stem cell’s sensitivity to endogenous epigenetic agents (hormones, cytokines, and growth factors).

However, regardless of how a mutation might occur in the somatic stem cells of any organ (brain, lung, liver, breast, prostate, kidney, skin, *etc.*), once adult stem cells have a mutation in a few critical genes that might affect the stem cell’s ability to divide “asymmetrically” [49], this stem cell will be unable to terminally differentiate. “Initiation” is then defined as a stem cell’s inability to divide asymmetrically under normal conditions. In this state, that “initiated” stem cell can and does exist in all of our organs because it can be suppressed by surrounding and communicating normal cells, either by negative secreted growth regulators or, if the stem cell has already been induced to partially differentiate (“Oncology as partially blocked ontogeny” [73]) but is “contact inhibited,” by surrounding normal gap junction functioning progenitor or differentiated cells [77]. The organism could live out its life without these “initiated” cells ever acquiring all the “hallmarks of cancers” [78,79].

**Figure 6:**
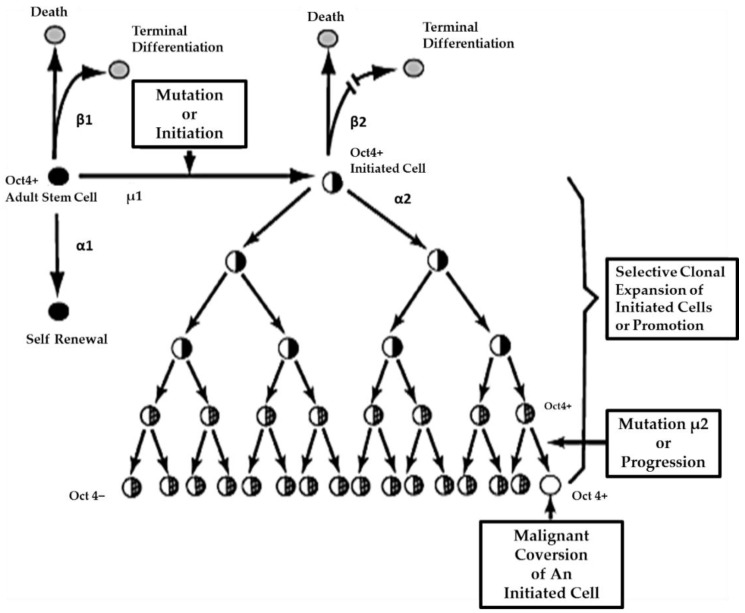
In this diagram, a normal adult stem cell is shown dividing asymmetrically to form one daughter that is committed to ultimately, terminally differentiate. The other daughter is designated to be identical to its mother adult stem cell (Oct4+). If that adult stem cell is exposed to some condition that prevents asymmetrical cell division but does not suppress Oct4 expression, it is operationally an initiated cell. That is, if mitotically stimulated to divide, it divides symmetrically to form two initiated, non-terminally differentiated cells. Initiation is, then, defined as the process that prevents an immortal normal adult stem cell from terminally differentiating or becoming “mortal.” These adult initiated stem cells are still Oct-4 positive or benign cancer stem cells. As these initiated Oct4+ cells are stimulated to proliferate and resist apoptosis, the growing benign tumor microenvironment changes and some of these initiated Oct4+ cells can partially differentiate into cancer non-stem cells (Oct4 negative). Eventually, additional stable mutational or epigenetic events occur, provoking the benign Oct-4+ cancer stem cells to become invasive, metastatic cancer stem cells. Permission granted by Nova Science Publishers Credit Line Information. In: Trosko, J.E. [81].

On the other hand, if the initiated cell is exposed to either endogenous (hormones, cytokines, or growth factors) or exogenous (drugs, pollutants, food, physical factors) epigenetic agents at or above threshold levels, chronically at regular exposures, in the absence of antioxidants [80] that can reversibly inhibit gap junctional intercellular communication between the initiated cell and surrounding normal cells, then these initiated cells can escape “contact inhibition” and proliferate but not terminally differentiate. Under these “promoting” conditions, papillomas in the skin, enzyme-altered foci in the liver, polyps in the colon, and nodules in the breast can form. These “initiated” cells form non-differentiated functions in any organ and populations of initiated stem cells that can be at higher risk of additional mutations and epigenetic changes needed to complete the carcinogenic process to become invasive and metastatic (Figure 6).

This process of promotion involves the “initiated” organ-specific adult stem cell and cell-cell communication. It is the process of initiation in an adult organ-specific stem cell that prevents its ability to undergo normal asymmetric cell division when exposed to some exogenous signal, such as some extracellular matrix molecule or the signal transducing elements associated with that extracellular molecule or some extracellular adhesion molecule.

**Figure 7:**
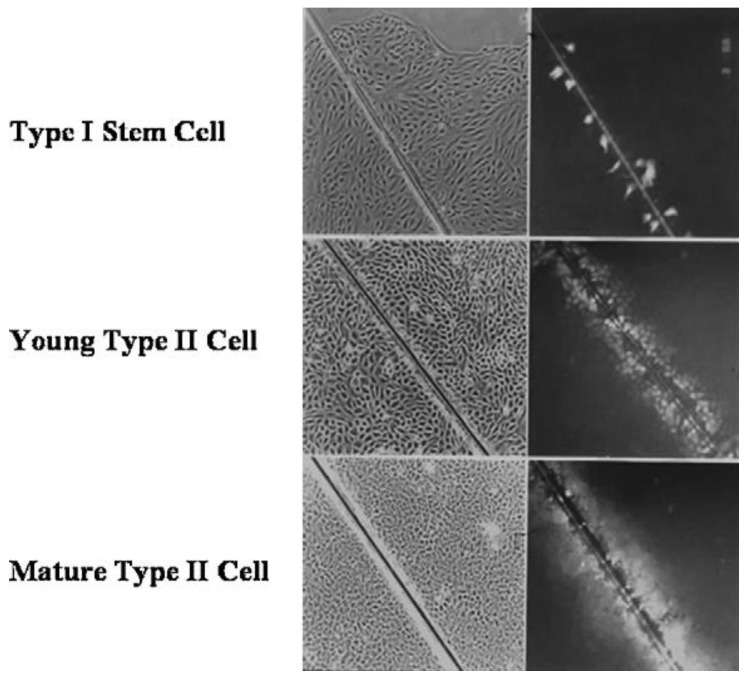
Using the scrape loading/dye transfer technique to measure GJIC by the diffusion of Lucifer yellow through functional gap junctions, these photographs illustrate that normal human adult breast stem cells do not have functional GJIC, while those human breast epithelial cells derived from the breast stem cells do have functional gap junctions. Permission granted from Trosko, J.E. and Chang, C.-C. [22].

**Figure 8:**
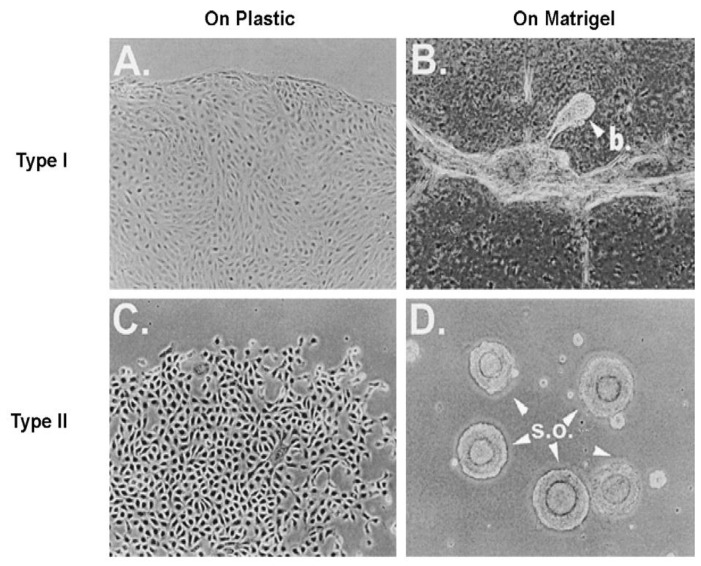

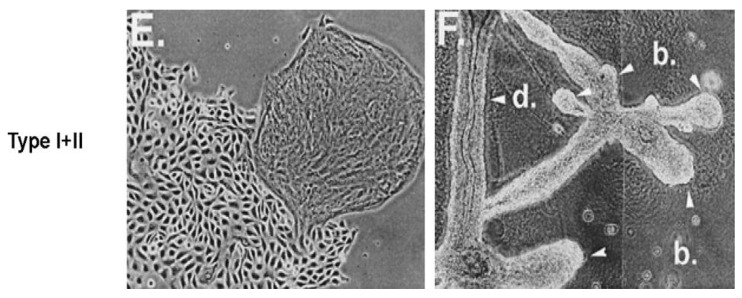
Human breast epithelial cell (HBEC) colonies on plastic and organoids on Matrigel formed from two types of normal HBECs. Type 1 and Type II colonies developed on plastic ((**A**,**C**), respectively) are morphologically distinguishable. On Matrigel, Type II cells typically formed a spherical organoid (s.o.) (**D**), whereas Type I cells formed a limited number of bud-like (**B**) structures and ascini. The combination of Type I and Type II cells ((**E**); two types of cells on plastics) in 1:2 or 1:3 ratios can generate many budding (**B**) ductal (**D**) structures in Matrigel (**F**) in 2–3 weeks. Credit to Sun *et al.* [82].

**Figure 9:**
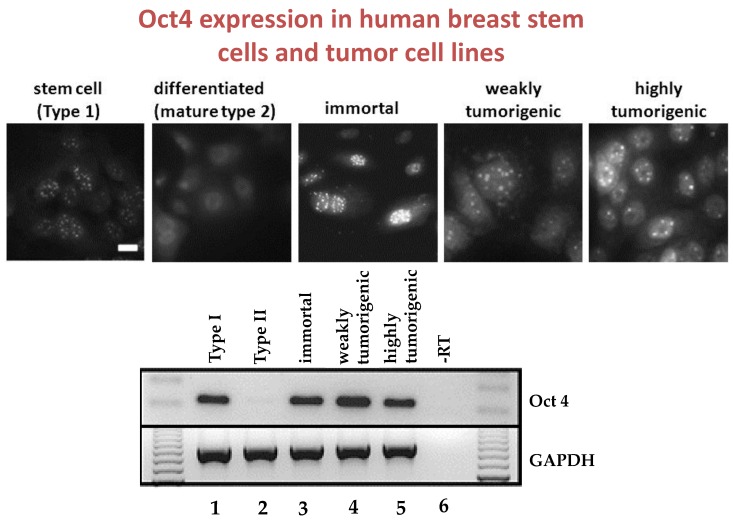
The composite of these figures illustrated that (a) the clonally derived normal human adult breast stem cells expressed Oct4+ or Type 1 via immuno-histochemical use of fluorescent antibodies to Oct4 in the normal breast stem cells, but not in the differentiated breast cells, still expressed in SV40 immortalized normal breast stem cells that were not tumorigenic, still expressed in the irradiated and weakly tumorigenic breast stem cells, and expressed in the neu/ErB-2 highly transformed clone. The RT-PCR data correlated with the immune-histochemical data of Oct4 in this series of human breast adult stem cells. Type 2 or differentiated Type 1 cells were derived from Type 1 or adult human breast stem cells after the Type 1 cells were exposed to cholera toxin (Kao *et al.* [83]). Permission granted by Karger Publishers.

An example that seems to be consistent with this hypothesis is shown in a series of figures (Figure 7, Figure 8 and Figure 9).

**Figure 10:**
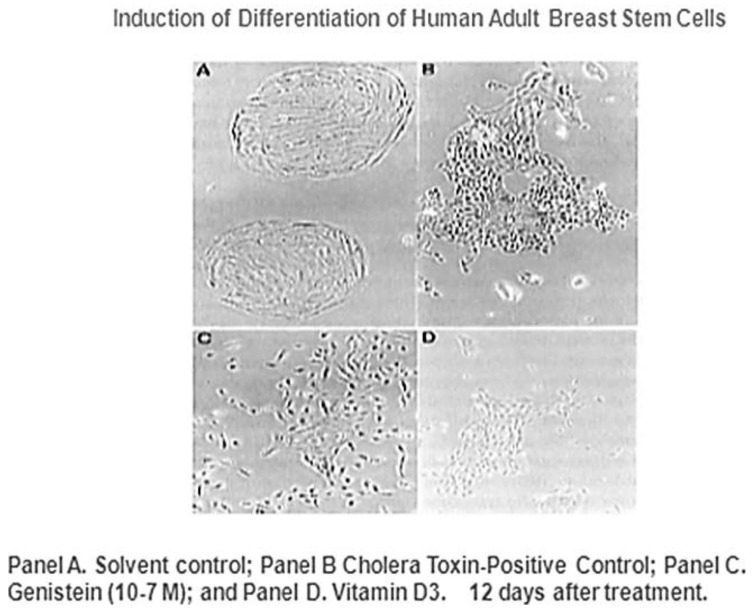
These four panels illustrate the induction of differentiation of Type I (human adult breast stem cells) to Type II (differentiated human breast epithelial cells, HBEC) by genistein and vitamin D3. The cells were treated with (**A**) ethanol (0.3%. *v/v*), solvent control; (**B**) cholera toxin (i ng/mL), positive control; (**C**) genistein (10.7 M); and (**D**) vitamin D, (10.9 M). Photos were taken 12 days after treatment. From Hsieh and Chang, [84]; Permission granted from Chinese Pharm, J. (Taiwan).

Since it has been shown that there are endogenous and exogenous chemicals that can block cell-cell communication, it would be logical to predict there would be agents that could either enhance cell-cell communication or interfere with other agents that block cell-cell communication. In Figure 10, it has been suggested by epidemiological studies that breast cancer can be prevented by several agents.

Given that breast cancer has been postulated to have originated from human breast stem cells [76], which do not have gap junctional intercellular communication, this demonstrates that cholera toxin, genistein, and Vitamin D3 cause these stem cells to differentiate [84]. If the developing female fetus has diet, drug, or environmental exposures that causes her breast stem cells to prematurely differentiate *in utero*, later in life, when her hormones are expressed during puberty, there are no organ-specific stem cells from which to make breast tissue, let alone be targets for breast cancer, so the risk of breast cancer would be reduced [85]. To generalize from this explanation, by increasing or decreasing organ-specific adult stem cells, one could increase or decrease the risk for cancer later in life. This would be a specific example of how cultural evolution, via changing nutrition/diets, could influence the biological evolutionary processes, leading to the development of the breast to influence breast cancer frequencies.

One of the “new” concepts in the understanding of carcinogenesis has been the introduction of the “cancer stem cell” or “cancer-initiating” cell [86,87,88,89,90,91,92,93,94,95,96,97,98,99,100,101,102,103,104]. It has come out of the recent demonstration that all cancers are a mixture of “cancer stem cells,” “cancer non-stem cells,” normal stromal cells in and around the tumor, and invasive inflammatory cells in some cases. It can be argued that, from the “stem cell” hypothesis of cancer, this “new concept” is what would be expected from that old hypothesis. From the perspective of prevention and treatment of cancer, within this initiation/promotion/progression model of human cancers, it would seem that prevention of the “initiating” event might be the most efficacious means of preventing cancers. Unfortunately, while it is possible to reduce exposure to agents that might mutate or initiate adult stem cells (one needs to not spend excess amounts of time in the sun), *one can never reduce to zero levels an initiating event in the stem cells.* However, given that the promotion of that “initiated” stem cell might take decades, in the case of adult cancers, to acquire all the “hallmarks of cancer” by exposures to epigenetic agents at or above threshold levels, in the absence of “anti-promoters,” at regular and chronic exposures the promotion phase would be the most efficacious phase during which to prevent cancers.

Cigarette smoking acts as a tumor-promoting condition [105]. In the addicted smoker, the condition is exactly what is needed for threshold and regular chronic exposure for the promotion of any initiated lung stem cell. Lung cancers can and do occur in non-smokers because initiation of lung stem cells can occur by normal “errors of stem cell replication” by many agents to which those individuals might be exposed that can cause chronic inflammatory stimulation of those initiated stem cells. Mutations in an oncogene found in lung cancer cells of smokers and non-smokers exhibit the same molecular mutation spectrum [106]. This implies that the initiated cells of both the smoker and non-smoker were “caused” by the same spontaneous errors of DNA replication, but promoted by different epigenetic agents (cigarette smokers in the one case; unknown promoters in the non-smoker’s case).

If one assumes that the promotion phase is the rate-limiting phase of the multi-stage, multi-mechanism process of human carcinogenesis, strategies to prevent cancer intervene with some factors that could cause the initiated cells either to be clonally expanded or prevented by some regular, chronic human activity. The obvious human activity that meets this criterion is eating. Food is nothing more than chemicals, some of which are nutrients, vitamins, minerals, fibers, natural or synthetic, supplements, physical, biological contaminants, and products of preparation. Since we normally eat every day, these food-related chemicals could stimulate the expansion of these initiated cells and act as tumor promoters, while others could do exactly the opposite and act as nutritional/dietary cancer chemopreventive agents.

**Figure 11:**
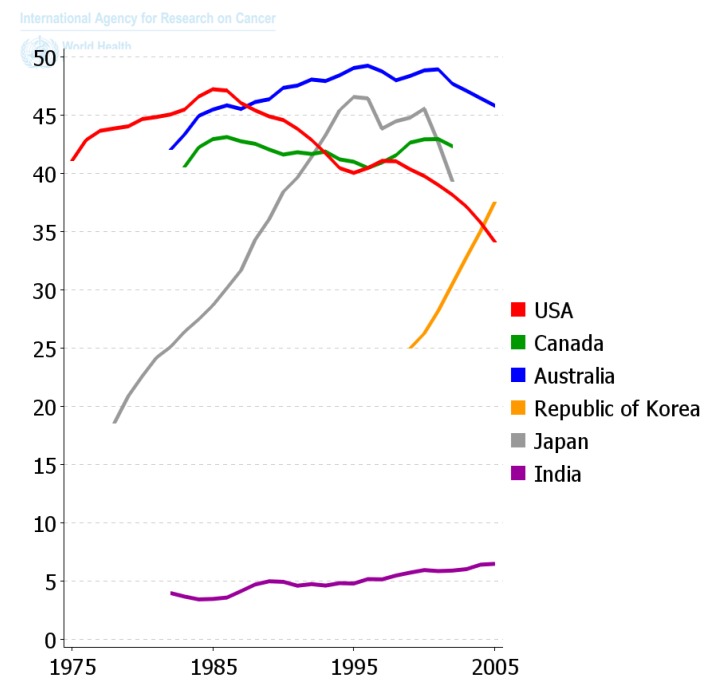
This figure illustrates the close correlation between the consumption of red meat and the frequency of colon cancer in different countries over 30 years. (The Y axis indicates frequencies of colon cancer per 100,000; the X axis indicates the years in which the frequencies were measured.) This clearly demonstrates, in the case of the Japanese and Korean incidences of colon cancer, that the “genetic background” of these two groups of people has little to do with the dramatic change in colon cancer. More likely, the changing diet in those countries is the driving force for the incidence of colon cancer. From: zur Hausen, [110]. Permission Granted.

Since the diaspora of human beings out of Africa and the long settlement of these ethnic groups in unique environments and the foods they supported, those ethnic groups that survive acquired the genes needed to benefit survival and reproductive potential. Only within recent decades has the diaspora of people and of foods, as well as new means of food preparation, created this collision of the biological and cultural factors related to diet that is creating all the problems related to global “metabolic diseases”. One can see in Figure 11 how powerful nutrition/diets are in modulating one chronic disease, namely colon cancer. When one plots the frequency of colon cancer in countries that are associated with the availability of red meat over the last 50 years or so, a reasonable conclusion can be made that the changes in diet have made a major impact on the frequency of this cancer. The genetics of ethnic populations, while not unimportant, is not the prime determinant, but the cultural nutritional/dietary environment is playing a major role. While the association of diets and cancer is not new, the point being made here is that eating foods every day, like smoking cigarettes every day, affects the promotion phase of human carcinogenesis. It can be a positive factor in preventing the appearance of cancer as well as many chronic diseases [2,49,57,107,108,109], or a negative factor in stimulating a higher risk. The complicating issue is that only rarely does one eat the same foods throughout a whole life. Moreover, even if one did eat the same foods each day, exposures to other sources of chemicals, e.g., drinking, smoking, medication, pollutants, as well as developmental changes in endogenous chemicals, could act additively, antagonistically, or synergistically to modify the impact foods could have on disease frequencies.

## 6. Senescence and Aging: The Price We Pay for the Evolutionary Appearance of Stem Cells and Differentiation

One of the complex problems in biology is determining if the aging process is independent of or directly responsible for the diseases associated with aging [111,112]. Could it be that the concepts of aging, diseases of aging, and cellular senescence involve very different mechanisms of causation; or do they share common mechanisms [69,77,113,114,115,116,117]? Without question, in the case of human beings, we know that there are genetic, environmental, and dietary factors that influence aging and diseases of the aging processes. Therefore, “Should aging be conceptualized as a disease?” An attempt to find answers to these questions would distract from the main objective of this *Commentary*, but these questions could provide some unifying insight into the role of stem cells in both aging and certain age-related disease processes, such as cancer. From the standpoint of making a distinction between aging and cellular senescence, the concept of aging usually relates to the breakdown of various homeostatic mechanisms of the whole intact organism. Cellular senescence has been conceptualized as a cell that seems to have lost its ability to replicate indefinitely [118].

In the case of normal human aging, our organs do not “age” uniformly. In other words, if an individual exposes him/herself to excessive amounts of UV light from the sun, particularly if one is less pigmented, the skin exhibits signs of aging (wrinkles; abnormal pigmentation), as well as enhanced risk to various kinds of skin cancers. If one smokes too many cigarettes, the lungs develop various dysfunctions of lung function (emphysema, *etc.*), as well as increased risk for lung cancers. Those who drink too much alcohol can develop various dysfunctions of the liver, such as cirrhosis of the liver; at the same time they put themselves at higher risk of liver cancer. In each of these cases, aging of the specific organ is correlated with risk to cancers in that organ.

In each of these examples, the environmental agent (UV light; chemicals in cigarette smoke; alcohol) usually targets a few organs of the body, while not affecting other organs. In other words, environmentally-targeted organs “age” or lose their normal homeostatic role within that organ but also their other whole-body homeostatic functions. Albino humans would have dramatically “aged” skin and eye function after ultraviolet light exposures to the sun, while their internal organisms will not have aged as dramatically. An alcoholic might have unaffected skin, while the liver would have dramatically aged. In the same fashion, a smoker might have non-functioning or aged lungs, while at the same time several of his/her organs might not have aged as rapidly as the lung.

Given that cancers in each of these environmentally-induced premature aged organs originated from a single organ-specific stem cell, there now seems to be a link between these organ-specific stem cells and aging.

There exist several human pre-mature “aging” syndromes, which could provide some insight into this uncertain link between aging and diseases of aging. While syndromes such as Hutchinson-Gilford progeria, Werner’s, and Cockayne’s affect very different aspects of aging, the progeria syndrome probably provides the best insight into the roles of stem cells and diseases of aging. It has recently been demonstrated that the individual with progeria inherits a mutation in a gene that affects stem cells’ ability to survive after exposure to oxygen after birth [119,120]. *In utero*, due to low oxygen tension compared to after birth, the development of the progeria embryo/fetus is relatively normal. Only after birth is there a dramatic interference in all organ growth. Because individuals with progeria do not survive for very long and the normal carcinogenic process takes many decades, the frequency of cancers in this rare syndrome is very low. Again, because of the small numbers of individuals with progeria, childhood cancers might be too scarce to determine whether the genetic mutation lowers the number of organ-specific adult stem cells so as to lower the risk of childhood cancers, as well as the endogenous growth factors or hormones needed to promote the initiated stem cells into metastatic cancers. Even in the case of Werner syndrome [121,122,123,124], disruption of the WRN gene in the embryonic stem cell resembles aspects of aging but only after differentiation into mesenchymal stem cells.

Another genetic syndrome, Down’s syndrome, is associated with birth defects, predisposition to leukemia, diabetes, cardiovascular diseases, premature aging, and—in those who live for an extended length of time—high risk for Alzheimer’s [125]. While this syndrome is genetic as defined by the inheritance of 3 × chromosome 21, the pathological consequences of inheriting three sets of normal genes is, in all likelihood, due to “epigenetic” mechanisms because of an imbalance of three sets of genes.

Another question is: “Could adult organ-specific stem cells ‘age’?” Normally, the organ-specific stem cell seems to have been evolutionarily selected to remain in a “quiescent state” to protect its genome from mutagenic events due to “errors in DNA replication.” Only in times of developmental needs or loss of massive numbers of progenitor and differentiated cells are these quiescent stem cells triggered into proliferation or “compensatory hyperplasia.” There seems to be some evidence of stem cells aging [126]. The redox state plays a major role in stem cells’ ability to either remain in a quiescent state or differentiate/undergo apoptosis [47,49,126,127].

Finally, if senescence, such as replicative-related stoppage of cell proliferation, first described by Hayflick [118], has been interpreted as an evolutionary-derived mechanism developed to prevent cancers [128], then a recent, very important observation should challenge our thinking about cellular senescence. Choi *et al.* [129] observed that putting replicative-induced senescent cells on the extracellular matrix, secreted by young cells, restored their ability to resume proliferation. This observation strongly suggests that senescence is an induced and reversible epigenetic phenomenon rather than an irreversible, mutagenic process. If this interpretation is correct, this phenomenon of “senescence” has implications, not only for the concept of aging, but also for the field of stem cell research, where senesced cells can be “reprogrammed” to be restored to a proliferative state [130].

In summary, aging and diseases of aging appear to represent opposite sides of the same “coin,” namely organ-specific stem cells. If, after exposure to environmental agents, the stem cell is stimulated to proliferate, thereby increasing the risk of an “error of DNA replication” or “error of DNA repair,” causing either a point mutation to initiate the stem cell or cell death to act as a tumor promoter [131], as in the xerodermatic pigmentation example, then cancer can follow. On the other hand, if an inherited mutation, as in progeria, causes death of organ-specific stem cells, then aging of the whole organism would occur. In other words, if a mutation leads to an organ-specific stem cell being prevented from asymmetric cell division or differentiation, a cancer can follow. If this hypothesis is correct, stem cell survival can be a target for several “diseases of aging” such as cancer, whereas stem cell death might lead to either whole body aging, as in the progeria example, or organ-specific aging, as in various environmentally induced causes of organ-specific stem cell death [132]. Cellular senescence, on the other hand, appears to be an environmentally induced epigenetic phenomenon (a normal “differentiation-like” process that can affect the organism’s physiological function, but that is both preventable and potentially reversible).

Yet cellular senescence seems to play roles in both aging and other diseases of aging. Radiation-induced senescence has been associated with stimulation-surviving cancer stem cells [133]. In addition, senescent cancer-associated fibroblasts secrete active MMP-2, which promotes keratinocyte dis-cohesion and invasion [134]. On the other hand, cellular senescence is causally implicated in generating age-related phenotypes and that removal of senescent cells can prevent or delay tissue dysfunction and extend health span [135].

## 7. Use of Human Cancer Stem Cells in 3D Organoids to Screen for Epigenetic Agents that Can Stimulate or Inhibit Their Growth

One of the modern consequences of biological evolution of cell communication led to the creation of cultural evolution. In brief, this long and complex history brought on the human global health problem by, ironically, allowing more human beings to live longer only to be plagued by increases in the “metabolic diseases” of diabetes, cardiovascular diseases, and cancer. While the patterns of these diseases varies from culture to culture, in the case of cancer where the picture is very clear, the frequency of cancers suggests about one in 4 people will be diagnosed with a cancer before death. This suggests (apart from the relatively rare cases of hereditary cancers) that external factors such as diet and lifestyle behaviors play a major role in modulating the appearance of cancer.

**Figure 12:**
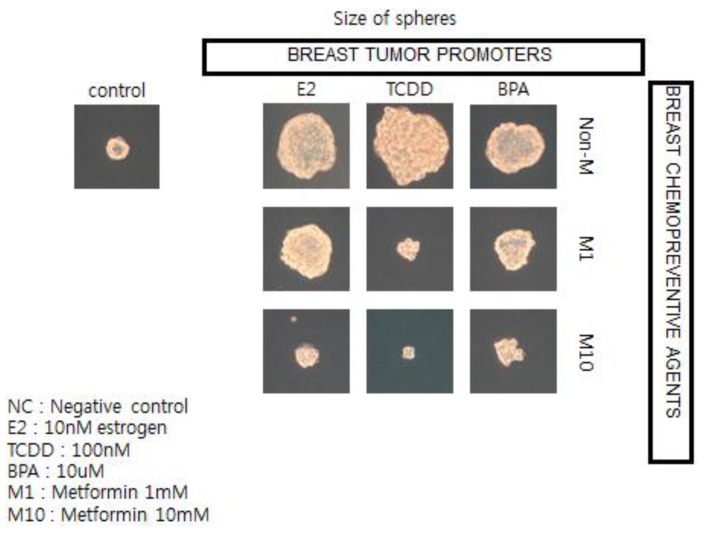
This figure illustrates MCF-7 human breast carcinomas cells expressing the estrogen receptor and forming mammospheres after exposure to a solvent, estrogen (E2), the epigenetic tumor promoter TCDD, and the epigenetic toxicant bisphenol A, with or without exposure to metformin (BPA). It is clear that E2, TCDD, and BPA cause enhanced growth of the mammospheres compared to the control mammospheres, while co-treatment with two concentrations of non-cytotoxic metformin significantly inhibited the growth stimulation of E2, TCDD, and BPA. Permission granted from PLOS ONE. In: Jung, J.-W., *et al.* [136].

To illustrate this point, and the possible linkages of these chronic diseases (diabetes, cardiovascular diseases, and cancer), because they all share underlying mechanisms (stem cells as target cells; cell communication being involved in their pathogenesis [57]; and the central role of chronic inflammation in their pathogenesis [70]), the observation that epidemiologists have made linking the associated usage of the type 2 diabetic treatment medication, metformin, with reduced risk of diabetes-associated cancers of the breast, liver, and pancreas stimulated the use of human breast cancer stem cells in a 3D “mammosphere” *in vitro* assay to screen for both potential or known human breast tumor promoters (estrogen; TCDD, and bisphenol A) and potential anti-cancer agents, such as metformin (see Figure 12) [136].

In brief, if these *in vitro* results showing how a natural compound, metformin, could interfere with the growth of human breast cancer stem cells stimulated by both natural and synthetic chemicals, are telling us something not only about the mechanism of cancer but about culture, we now have a logical link between gap junctional intercellular communication and stem cells, leading to the abstract thinking, language, and technology that have allowed human beings to create a culture that allows us to live long enough to get cancers—in part due to processes beyond our control and in part through actions that we can control.

Because one of the highest order differentiated functions of human stem cells and cell to cell communication has been the emergence of the “mind” from the organization of neurons, derived from specific adult stem cells in the brain [137], there cannot be any denial that adult brain stems exist in the brain and that alterations in cell communication by chemicals can affect gap junctional communication in neuronal cells (alcohol; phenobarbital, DDT, caffeine; *etc.*). Therefore, much of what we have “consciously” done with our ability to use our communication abilities has been to create the cultural conditions that have favored the induction of metabolic diseases by our many actions.

One additional link between the single-celled organism’s ability to survive in an anaerobic environment and the metazoan “cancer stem cell” surviving cancer treatment will be attempted [138]. When we look back at the history of the lethal microbial disease associated with tuberculosis (TG), we see how the discovery of so-called “wonder drugs” at that time led to hope that this devastating disease could be “cured” [139]. However, their short-lived success was met with the reality of a drug-resistant TB organism that appeared after treatment with these drugs and vaccines. A very similar historic event occurred with the non-infectious associated disease of cancer. After decades of searching for treatments (radiation, chemicals), many drugs were brought into clinical trials, some of which produced a small extension of life span, but none could ever be classified as a cure for cancer. In many, if not all, of these treatments, a transient shrinkage of tumors might be seen, but in time anti-cancer treatment resistance is observed. In both cases, early interpretation of the emergence of microbial or cancer resistance to treatment was that the treatment “induced” resistance in a few cells of the disease. Today, the explanation seems to be that these anti-disease treatments only select out of the original population cells that were naturally resistant due to spontaneous mutations.

**Figure 13:**
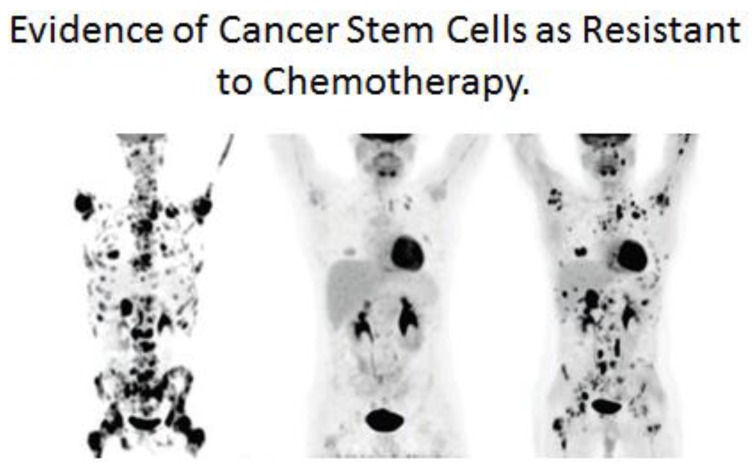
Dramatic but transient effect of new cancer treatment drug. Within two months, a novel drug candidate shriveled a man’s metastasized cancer (center). One month later, the cancer, now resistant, resurged. Permission granted from The New England Journal of Medicine; Rudin *et al.* [140].

In the case of the cancer stem cell, if it did arise from a normal adult stem cell that metabolizes via glycolysis, in a manner similar to an anaerobic bacterium, and expresses drug-transporter genes, it grows into a tumor. As it grows, it alters the microenvironment of the tumor, creating new conditions for some of these cancer stem cells to partially differentiate. When these tumors are detected, they are mixtures of cancer stem cells and cancer non-stem cells. Treatment of these tumors (see Figure 13), leads to immediate reduction of the cancer non-stem cells that are not resistant to current anti-cancer therapies. However, after the death of the cancer non-stem cells, the surviving cells are the cancer stem cells. These surviving cancer stem cells were not induced by the anti-cancer treatments, but were selected out of the mixed tumor population.

## 8. The Biological Evolution of Brain Stem Cells, Neurons, Cellular, and Mind Communication Can Lead to a Cultural Evolution of Global Quorum Sensing of Pluralistic Cultures

If microbial “quorum sensing” was one early step in the evolution of gap junctional intercellular communication, then it has been speculated that over millions of years the integrated communication mechanisms, together with stem cells, led to the emergence of *Homo sapiens* able to communicate with each other to create culture. Culture brought about new adaptive means to survive and reproduce and has created the global environment in which 7 billion of us are making a major impact on our quality of life [2]. Furthermore, this integrated communication system helps us to understand how homeostatic control of the proliferation, differentiation, and apoptosis of our cells can lead to normal development and health. By the same token, it demonstrates how the unprogrammed or disrupted homeostatic control of cell-cell communication can lead to a decline in body and brain functions.

From an evolutionary perspective, the fundamental problem facing all life forms today is the collision between the millions of years of selection for adaptive genes for survival and reproduction by biological evolutionary forces in a physically changing planet with the rapidly changing global environment caused by cultural evolutionary forces. Since all life forms, from bacteria to human beings, share a common dependence on different sources of food for energy and have similar homeostatic mechanisms for survival and reproduction, it is imperative that we understand how the various forms of cell-cell communication (from quorum sensing to global human quorum sensing) are affecting the health and survival of all forms of life.

**Figure 14:**
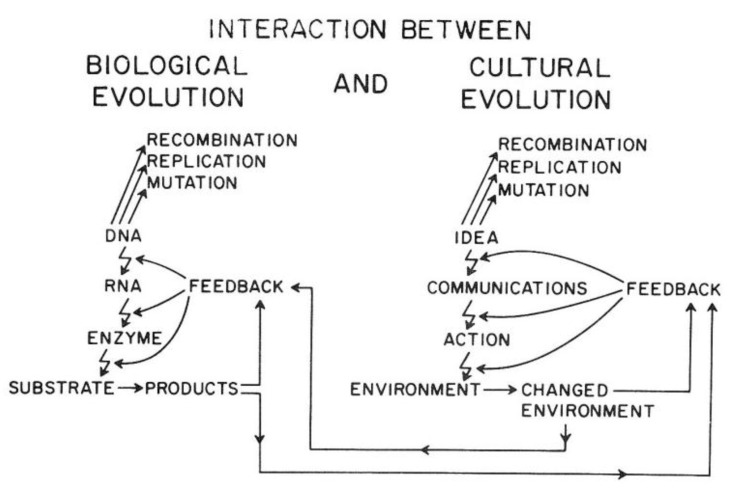
Feedback between processes of biological and cultural evolution (Modified from Figure 8, p. 107, in Potter’s Bioethics: Bridge to the Future; Prentice-Hall; New York; 1971. [141]).

Van R. Potter provided a conceptual framework showing how the ability of *Homo sapiens* to create new ideas acts to change the cultural codes for human survival in a similar manner to how random mutations and epigenetic changes alter genetic codes. Indeed, he shows how the two sources of creating new “information” affect each other. In brief, this overarching framework explains the direct link between biological and human evolution (see Figure 14).

As illustrated in Figure 14, the genetic information for both individual and species survival is coded in the DNA molecules. Either by mutations to change the genetic information in the genome or epigenetically by altering the expression of those genes, survival, health, and reproductive capacity can be either enhanced or diminished. In the pre-*Homo sapiens* planet, most of those mutational/epigenetic changes were caused by errors of DNA repair or by spontaneous errors of DNA replication of the single-celled organism or by the replication of stem cells in multi-celled organisms.

On the other hand, *Homo sapiens*, with his/her ability to create new ideas and transform them into things/action (create fire; domesticate animals, make tools, develop agricultural practice, “build abstract and concrete walls” [142]), had a powerful means to change the very environment in which all life forms have to adapt quickly.

In other words, *ideas* (existing and accepted social and cultural knowledge for survival) act as *mutagens* to alter the species’ genetic code for survival. Even more importantly, these cultural ideas can influence biological mutagens and epigenetic mechanisms. In principle, as with mutagens, the effect on survival depends on the context of the new idea. Without that context, one cannot judge the mutation/idea to be “good” or “bad”. Therein lies the problem, for the genetic/cultural change brought about by a new mutation or idea might have short-term benefits but long-term consequences, since future and inevitable changes in the physical/social/cultural worlds cannot be predicted by non-*Homo sapiens* organisms and are rarely accurately predicted by *Homo sapiens*. Furthermore, especially with cultural ideas or information, the information could be dead wrong on one hand, or just incomplete on the other hand.

One example of this collision of biological and cultural evolutionary interactions is global health issues, including increases in “metabolic diseases”, diseases caused by *in utero* exposure leading to birth defects and diseases later in life (the Barker hypothesis), and diseases of aging. In the latter case, these diseases of aging are, in part, the consequences of the beneficial application of new scientific ideas to reduce infant deaths caused by infectious disease (better sanitation, better nutrition, better food production, new medications, *etc.*), which allowed the human population to survive for longer. However, by living longer, we are now exposed to the “effluence of our affluence” [4]. To produce more food for the exploding human population, political/economic/legal/cultural policies have changed the physical global ecosystem (air, land, forest, water) to produce foods that provide calories but not necessarily the needed nutrients for health.

## 9. Conclusions

If we extrapolate from brain cell-cell communication to human consciousness and language, it might be plausible in the future to connect those important scientific mechanistic unknowns concerning how human consciousness emerges out of neuronal cell-cell communication, the leap from microbial quorum sensing to potential “global human quorum sensing” might not seem so far-fetched.

In the vast history of the evolution of living organisms, looking back at human beings’ very small place in that continuum one sees that, until very recently, there were only few means by which any human being could communicate with another human being with different language abilities. There was the telephone, telegraph, mail delivery, air and boat transportation, photographs, *etc*.

Only recently have desktop computers and Internet connections became available to most people. Within the last decade, cable TV, smart phones, and new social networking on a global scale, such as Twitter, Facebook, Instagram, *etc.*, became available. When one thinks about how long it takes for a new scientific concept, such as linking DNA with human heredity or the concept of evolution, to take hold in our understanding of human nature, even with all these new technologies these concepts have not yet achieved “global consciousness” via traditional educational means. Yet these new means of communication, created by our ability to differentiate neurons from stem cells, has allowed for selective and powerful means to have a “global quorum sensing.” One needs only examine how quickly and effectively terrorist-related recruitment can affect the collective behavior of some individuals, globally, via social media to see that the Internet can communicate ideological stimuli to affect behavior.

The diaspora of *Homo sapiens* to different physical environments helped to create multiple cultures. Each is based on different “world views of human nature” that shape the global pluralistic values that now govern the use (or misuse) the technology [7]. So, while the means to create a global “consciousness” or “global quorum sensing” is theoretically feasible, it seems that communicating the same (extracellular information) that can be shared almost instantaneously will not induce the same intracellular signaling to bring about the same neuronal response. The current pluralistic reactions to the issue of global warming suggest that, unlike a single-celled organism’s reaction to “quorum sensing” of an environmental stress, human beings’ “global quorum sensing” requires some evolutionary adjustments.
